# Reversal of epigenetic aging and immunosenescent trends in humans

**DOI:** 10.1111/acel.13028

**Published:** 2019-09-08

**Authors:** Gregory M. Fahy, Robert T. Brooke, James P. Watson, Zinaida Good, Shreyas S. Vasanawala, Holden Maecker, Michael D. Leipold, David T. S. Lin, Michael S. Kobor, Steve Horvath

**Affiliations:** ^1^ Intervene Immune Los Angeles CA USA; ^2^ UCLA Division of Plastic and Reconstructive Surgery David Geffen School of Medicine Los Angeles CA USA; ^3^ Departments of Microbiology and Immunology Stanford University Stanford CA USA; ^4^ Stanford Medical Center Stanford CA USA; ^5^ Institute for Immunity, Transplantation and Infection, Stanford School of Medicine Human Immune Monitoring Center Stanford CA USA; ^6^ Department of Medical Genetics, BC Children's Hospital Research Institute Centre for Molecular Medicine and Therapeutics, University of British Columbia Vancouver BC Canada; ^7^ Human Genetics, David Geffen School of Medicine University of California Los Angeles CA USA; ^8^ Present address: Stanford Cancer Institute Stanford University Stanford CA USA

**Keywords:** c-reactive protein, lymphocyte‐to‐monocyte ratio, naive T cells, PD‐1, PSA, thymic regeneration

## Abstract

Epigenetic “clocks” can now surpass chronological age in accuracy for estimating biological age. Here, we use four such age estimators to show that epigenetic aging can be reversed in humans. Using a protocol intended to regenerate the thymus, we observed protective immunological changes, improved risk indices for many age‐related diseases, and a mean epigenetic age approximately 1.5 years less than baseline after 1 year of treatment (−2.5‐year change compared to no treatment at the end of the study). The rate of epigenetic aging reversal relative to chronological age accelerated from −1.6 year/year from 0–9 month to −6.5 year/year from 9–12 month. The GrimAge predictor of human morbidity and mortality showed a 2‐year decrease in epigenetic vs. chronological age that persisted six months after discontinuing treatment. This is to our knowledge the first report of an increase, based on an epigenetic age estimator, in predicted human lifespan by means of a currently accessible aging intervention.

## 1 INTRODUCTION

Population aging is an increasingly important problem in developed countries, bringing with it a host of medical, social, economic, political, and psychological problems (Rae et al., 2010). Over the last several years, many biomedical approaches to ameliorating aging have been investigated in animal models, and some of these seem able to reverse general aspects of aging in adult mammals based on a variety of physiological measurements (Das et al., 2018; Ocampo et al., 2016; Zhang et al., 2017). However, to date, evidence that systemic aging can be reversed has not been substantiated by determinations of epigenetic age, which can now provide a simple but compelling indication of biological as opposed to chronological age (Horvath & Raj, 2018; Jylhava, Pedersen, & Hagg, 2017). In addition, there is a need to specifically address immunosenescence stemming from thymic involution (Bodey, Bodey, Siegel, & Kaiser, 1997). Thymic involution leads to the depletion of critical immune cell populations (Arnold, Wolf, Brunner, Herndler‐Brandstetter, & Grubeck‐Loebenstein, 2011), resulting in a collapse of the T‐cell receptor (TCR) repertoire in humans after the age of ~63 (Naylor et al., 2005), and is linked to age‐related increases in cancer incidence (Falci et al., 2013), infectious disease (Ventevogel & Sempowski, 2013), autoimmune conditions (Goronzy & Weyand, 2003), generalized inflammation (Goronzy & Weyand, 2003), atherosclerosis (Dai, Zhang, Wang, Wu, & Liang, 2018), and all‐cause mortality (Fernando‐Martinez et al., 2013; Roberts‐Thomson, Whittingham, Youngschaiyud, & Mackay, 1974; Strindhall et al., 2007). In contrast, maintained immune function is seen in centenarians (Strindhall et al., 2007). Although thymic function in aging also depends on the supply of T‐cell progenitors from the bone marrow, which declines in relation to the output of myeloid HSCs with age (Akunuru & Geiger, 2016), the net number of lymphoid precursors does not change with age (Montecino‐Rodriguez et al., 2019), and migration of T‐cell precursors from the bone marrow also appears to depend on thymic function (Haar, Taubenberger, Doane, & Kenyon, 1989).

For these reasons, we conducted what may be the first human clinical trial designed to reverse aspects of human aging, the TRIIM (Thymus Regeneration, Immunorestoration, and Insulin Mitigation) trial, in 2015–2017. The purpose of the TRIIM trial was to investigate the possibility of using recombinant human growth hormone (rhGH) to prevent or reverse signs of immunosenescence in a population of 51‐ to 65‐year‐old putatively healthy men, which represents the age range that just precedes the collapse of the TCR repertoire. rhGH was used based on prior evidence that growth hormone (GH) has thymotrophic and immune reconstituting effects in animals (Kelley et al., 1986) and human HIV patients (Napolitano et al., 2008; Plana et al., 2011). Because GH‐induced hyperinsulinemia (Marcus et al., 1990) is undesirable and might affect thymic regeneration and immunological reconstitution, we combined rhGH with both dehydroepiandrosterone (DHEA) and metformin in an attempt to limit the “diabetogenic” effect of GH (Fahy, 2003, 2010; Weiss, Villareal, Fontana, Han, & Holloszy, 2011). DHEA has many effects, in both men and women, that oppose deleterious effects of normal aging (Cappola et al., 2009; Forti et al., 2012; Shufelt et al., 2010; Weiss et al., 2011). Metformin is a powerful calorie restriction mimetic in aging mice (Dhahbi, Mote, Fahy, & Spindler, 2005) and has been proposed as a candidate for slowing aging in humans (Barzilai, Crandall, Kritchevsky, & Espeland, 2016). Neither DHEA (Riley, Fitzmaurice, & Regelson, 1990) nor metformin are known to have any thymotrophic effects of their own.

## 2 RESULTS

### 2.1 Treatment safety and side effects

**Figure 1:**
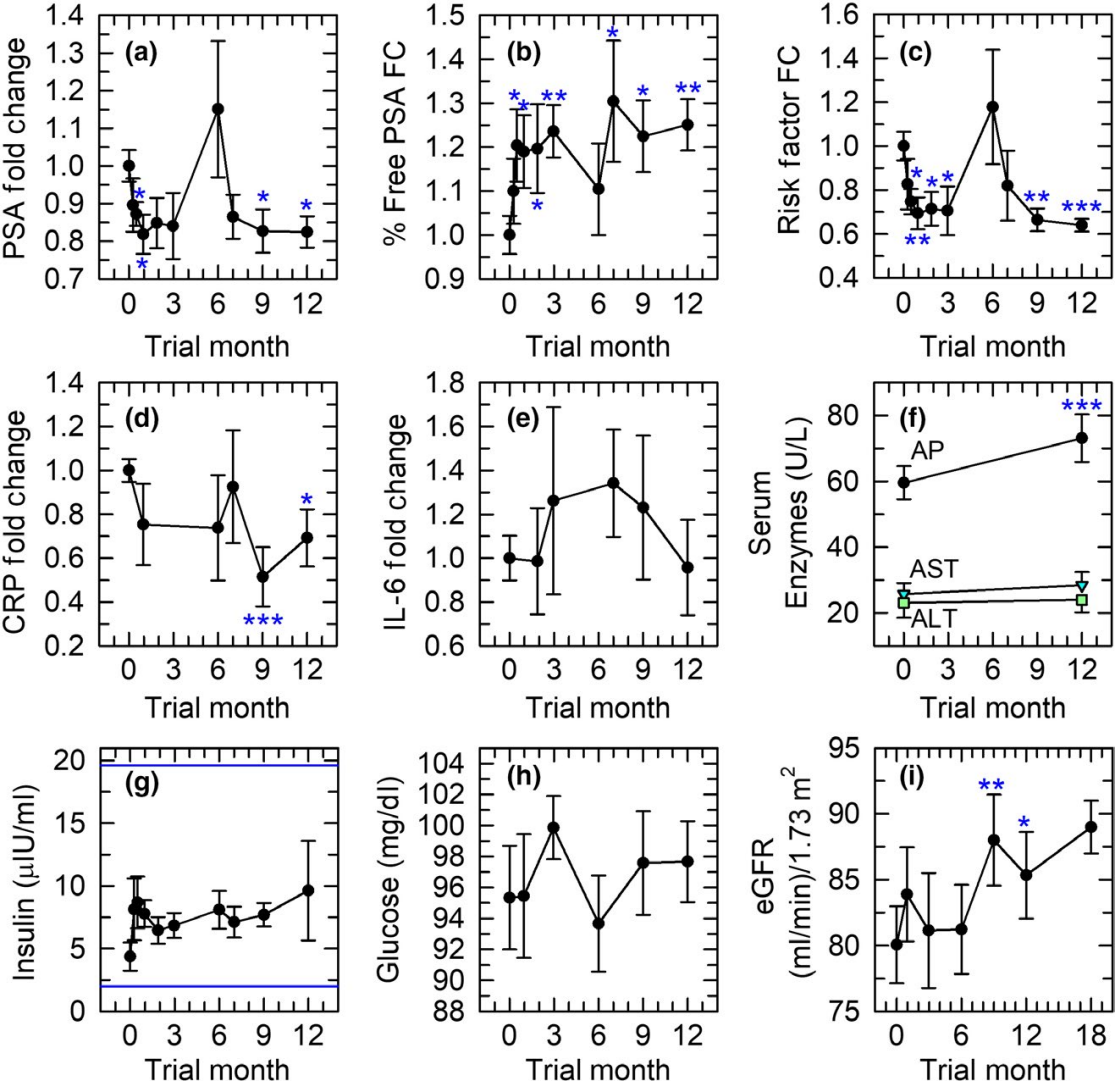
Treatment safety indices. In this and other figures, error bars depict SEMs; baseline SEMs for normalized data were obtained as described in Experimental Procedures. Asterisks denote *p* < .05 (*), *p* < .01 (**), and *p* ≤ .001 (***). (a) Prostate‐specific antigen (PSA). (b) Fold change (FC) in percent free PSA. (c) Fold change in the ratio of PSA to percent free PSA (“risk factor”), which rises as prostate cancer risk rises. (d, e) Pro‐inflammatory indices (c‐reactive protein (CRP)) and IL‐6). (f) Serum alkaline phosphatase (AP), aspartate aminotransferase (AST), and alanine aminotransferase (ALT). The increase in AP, while statistically significant, was quantitatively negligible and remained well within the normal range. (g) Maintenance of insulin levels within the upper and lower limits of the normal range (indicated by the horizontal lines). (h) Lack of change in serum glucose, which remained within the normal range. (i) Improvement in estimated GFR at 9 and 12 months of treatment, with a trend toward continued improvement 6 months after discontinuation of treatment

A primary concern in this study was whether increased levels of a mitogen (IGF‐1) might exacerbate cancerous or precancerous foci in the prostate. Both of these changes should be detectable by measuring PSA or percent free PSA levels. However, PSA, percent free PSA, and the ratio of PSA to percent free PSA, an overall index of prostate cancer risk, improved significantly by day 15 of treatment and remained favorably altered to the end of 12 months (Figure 1a–c). A brief spike in PSA at 6 months in two volunteers was rapidly reversed and, after volunteer consultation, was interpreted as reflecting sexual activity close to the time of PSA testing. No change in testosterone levels was observed.

Another significant concern was whether augmenting immune activity might exacerbate age‐related inflammation. However, CRP declined with treatment, the decline reaching statistical significance by 9–12 months (Figure 1d). The pro‐inflammatory cytokine, IL‐6, did not change (Figure 1e).

No remarkable changes were noted in serum albumin, lipids, hemoglobin, hematocrit, platelet count, electrolytes, and hepatic enzymes (Figure 1f). Insulin levels were in general adequately controlled by co‐administration of DHEA and metformin (Figure 1g) (although one outlier increased mean insulin at 12 months), and glucose levels did not change (Figure 1h). Finally, estimated glomerular filtration rates (eGFR), which are relevant to the potential for lactic acidosis with metformin as well as to treatment efficacy, showed a statistically significant improvement after 9–12 months (with a trend toward improvement at 18 months as well) (Figure 1i). Side effects were mild, typical of rhGH administration, and did not require dosing modification except in two cases. Side effects included arthralgias (2 cases), anxiety (1 case), carpal tunnel syndrome (1 case), fluid retention (1 case), mild gynecomastia (1 case), and muscle soreness (1 case). One trial volunteer was removed from the study after approximately one month due to self‐reported bradycardia, which preceded the trial, and belated admission of a strong familial history of cancer.

### 2.2 Thymic and bone marrow regenerative responses

**Figure 2:**
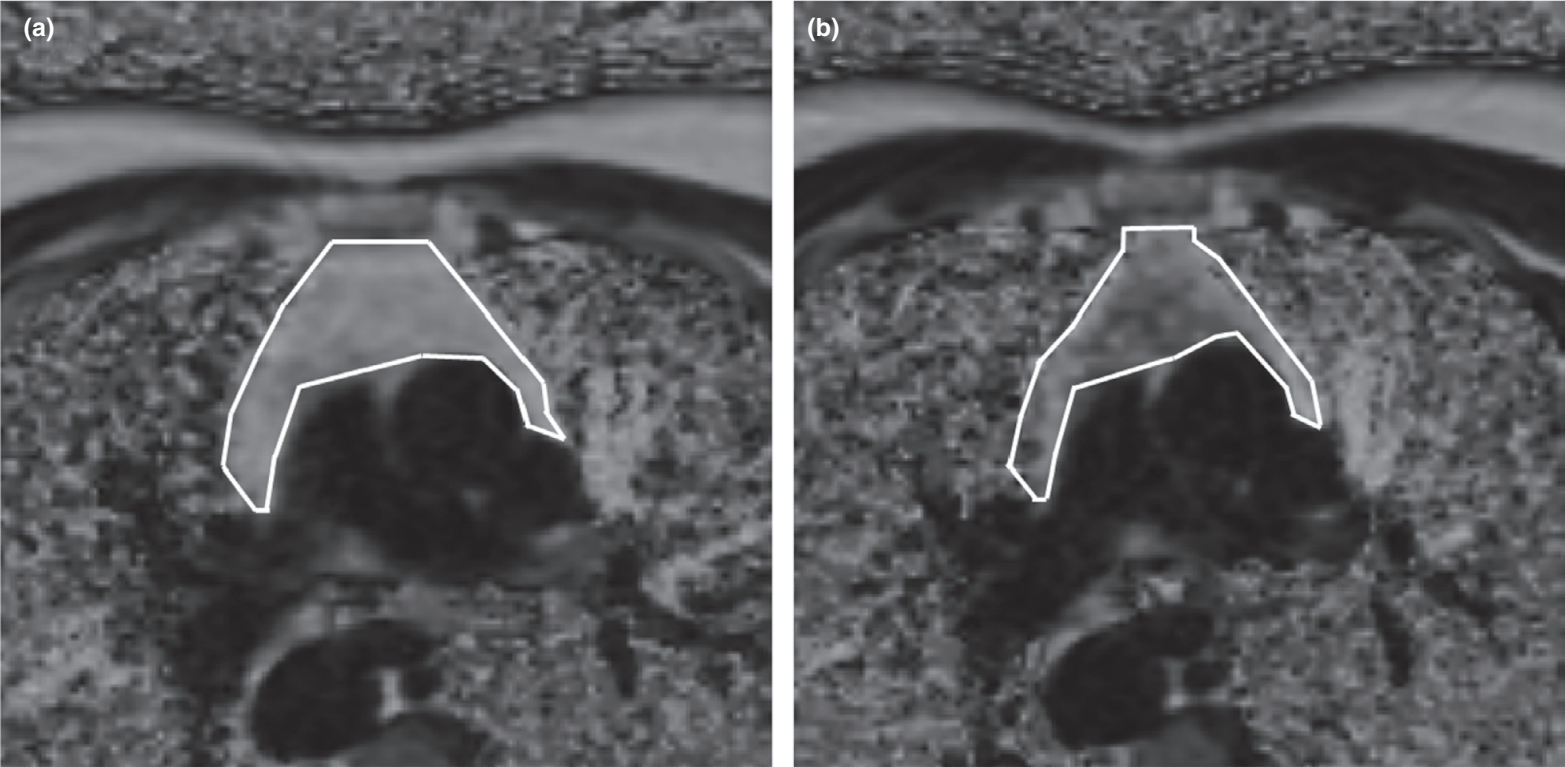
Example of treatment‐induced change in thymic MRI appearance. Darkening corresponds to replacement of fat with nonadipose tissue. White lines denote the thymic boundary. Volunteer 2 at 0 (a) and 9 (b) months

**Figure 3:**
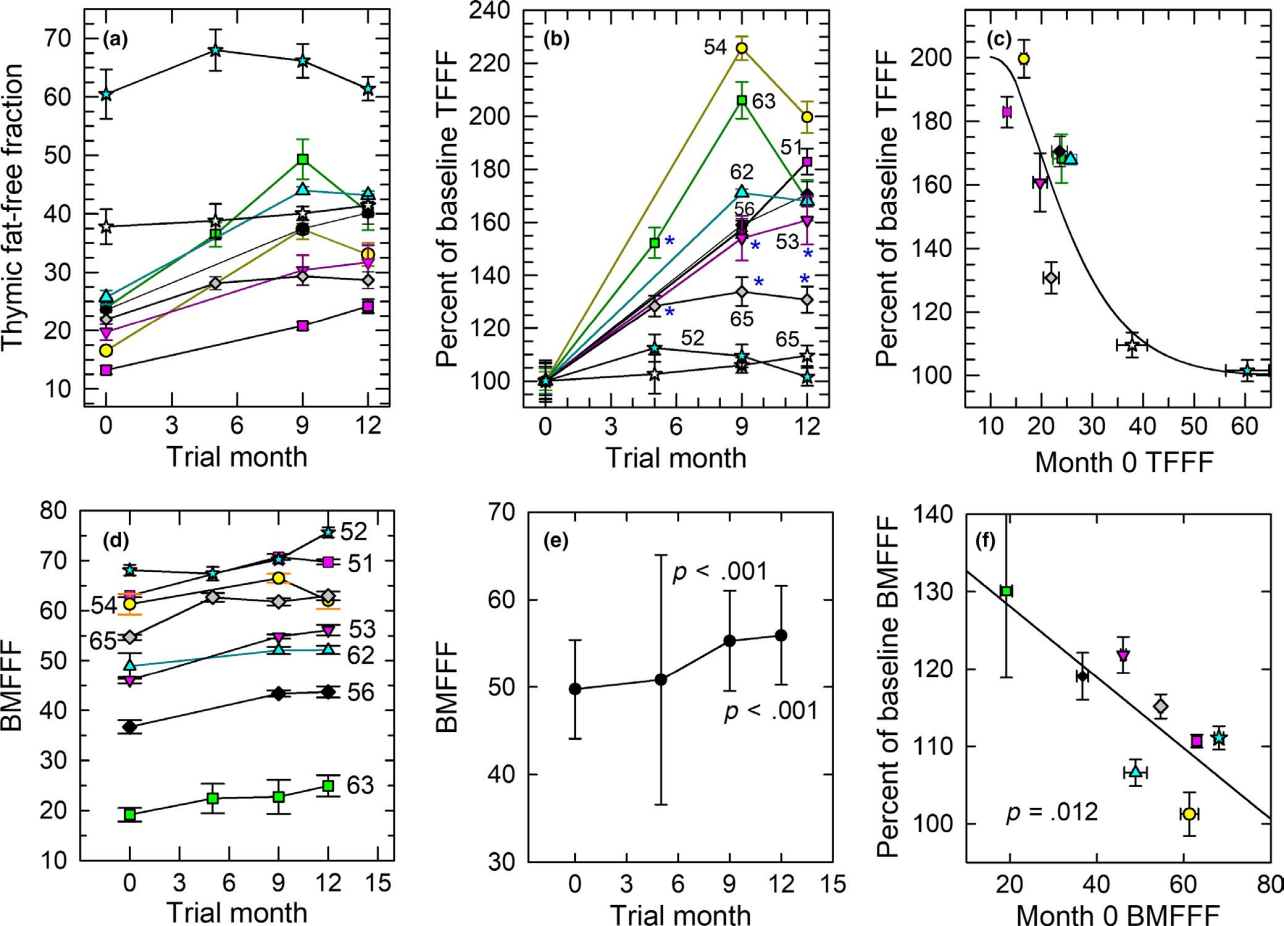
Quantitative MRI‐based regeneration outcomes. Like symbols denote the same individuals in each panel. (a) Individual absolute changes in TFFF. Statistically significant improvements are evident for each individual with the exception of two volunteers who showed high TFFF at baseline (stars); overall significance by linear mixed‐model analysis: *p* < 9 × 10^–17^ (see text). (b) Relative changes in TFFF for each individual. The age of each individual at trial entry (noted adjacent to each line) does not correlate with the magnitude of the depicted changes. The highest p values (*p* > .01) for significant individual responses are denoted with asterisks; for clarity, higher significance levels are not designated. (c) Sigmoidal dependence of TFFF change at 12 months on baseline TFFF, showing greater improvements for thymi with lower basal TFFFs (*p* < .007). (d) Individual changes in sternal bone marrow fat‐free fraction (BMFFF) (volunteer age noted adjacent to each line). (e) Mean overall changes in BMFFF. (f) Linear dependence of 12‐month BMFFF on basal BMFFF (*p* = .012), showing the largest relative changes in individuals with the lowest baseline BMFFFs. BMFFF data for one volunteer could not be evaluated

Obvious qualitative improvements in thymic MRI density were observed and are illustrated in Figure 2. Quantitatively, the overall increase in the thymic fat‐free fraction (TFFF) was significant at the *p* = 8.57 × 10^−17^ level based on linear mixed‐model analysis, implying a restoration of thymic functional mass. The improvements were significant in 7 of 9 volunteers (Figure 3a–c). Two volunteers had abnormally low levels of thymic fat (high TFFF) at baseline, and their TFFFs did not significantly improve with treatment (peak relative changes of +9.6% (*p* > .3) and +12.4% (*p* > .2); Figure 3b). Their lack of response was not age‐dependent. Instead, improvement in TFFF was dependent upon baseline TFFF, regardless of baseline age (Figure 3c).

By comparison, sternal BMFFF increased to a much lesser degree, but with such consistency (Figure 3d) as to reach high statistical significance (Figure 3e; *p* < .001 for single‐point comparison, or *p* = 9.5 × 10^−12^ for formal linear mixed‐model analysis). Bone marrow, similar to thymus, showed a pattern of increased BMFFF with increased baseline fat content, but the details of the pattern were different (Figure 3f). This difference plus the more robust replacement of thymic vs. bone marrow fat is consistent both with a specific reversal of thymic involution rather than generalized regression of body fat owing to GH administration and with possible stimulation of bone marrow T‐cell progenitor production by GH (French et al., 2002; Hanley, Napolitano, & McCune, 2005).

### 2.3 Immune cell subset and cytokine changes

**Figure 4:**
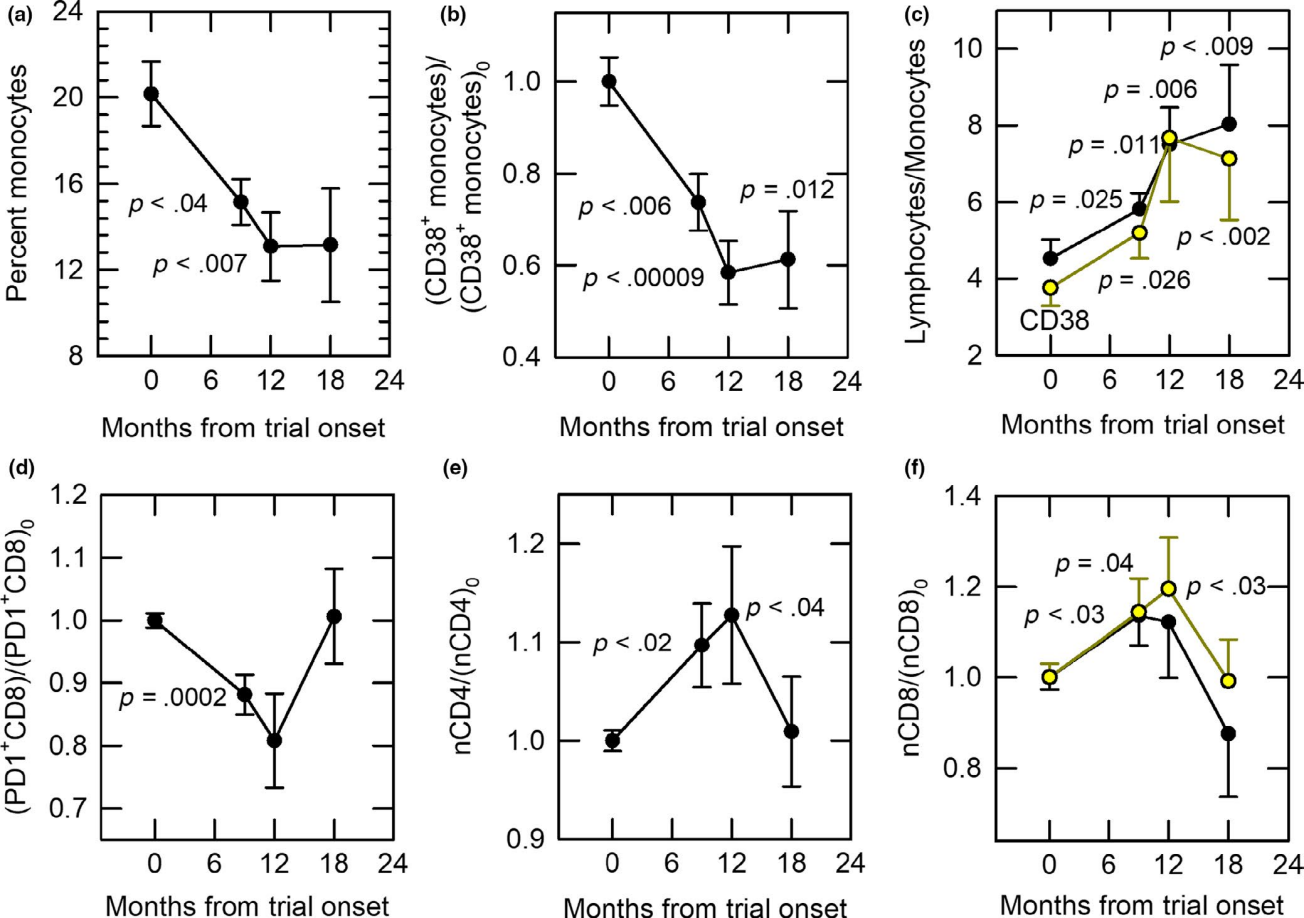
Immunological responses to treatment. (a) Decline (~35%) in monocyte (CD33^+^ cell) percentages with treatment. When normalized to baseline, the decline in monocytes remained significant even at 18 months (*p* = .022; Table S1). (b) Persistent decline (~40%) from baseline [(CD38^+^ monocytes)_0_] in CD38^+^ monocytes with treatment. (c) Persistent increase in the lymphocyte‐to‐monocyte cell ratio. Black points: lymphocyte‐to‐monocyte ratio; yellow points: lymphocyte‐to‐CD38^+^ monocyte ratio. (d) Decrease in normalized PD‐1‐positive CD8 cell percentage with treatment. (e) Increase in normalized naïve CD4 cells (nCD4) vs. baseline [(nCD4)_0_] with treatment. (f) Increase in normalized naïve CD8 cells with treatment. Black points: all volunteers (*p* = .03 at 9 months). Yellow points: all volunteers minus the most extreme outlier of Figure 3a–c (represented by blue stars in those figures): *p* = .04 at 9 months and *p* < .03 at 12 months

Analysis of CyTOF‐defined immune cell populations revealed the most robust changes to be decreases in total and CD38‐positive monocytes (Figure 4a, b) and resulting increases in the lymphocyte‐to‐monocyte ratio (LMR) (Figure 4c). Linear mixed‐model correlation analysis also showed a significant correlation after 9 and 12 months of treatment between TFFF and the reduction in CD38^+^ monocyte percentage (*r* ^2^ = .59, *p* = .01) as well as a correlation between TFFF and the ratio of lymphocytes to CD38^+^ monocytes (*r* ^2^ = .55, *p* = .018). Similar trends were seen for total monocytes vs. TFFF and for the overall LMR vs. TFFF (respectively, *r* ^2^ = .40, *p* = .039, and *r* ^2^ = .45, *p* = .019) when correlated across all treatment times (0, 9, and 12 months). The changes in mean monocyte populations persisted 6 months after discontinuation of treatment (*p* = .012 for normalized CD38^+^ monocytes and *p* = .022 for normalized total monocytes: Table S1), and the increase in LMR remained highly significant at 18 months as well.

PD‐1‐positive CD8 T cells declined significantly with treatment (Figure 4d). By gating on naïve T cells, we were able to detect significant increases in both naïve CD4 and naïve CD8 T cells (Figure 4e–f) and a significant (*p* = .017) increase in the percentage of CD31^+^CD45RA^+^CD4^+^ cells (CD4 recent thymic emigrants, or RTEs) over the course of treatment (linear correlation, Figure S1). We did not detect consistent changes in senescent T cells measured either as CD57^+^ cells or as CD28^‐^ cells. However, we did detect a significant increase in serum FGF‐21 levels (Figure S2).

### 2.4 Epigenetic age regression

**Figure 5:**
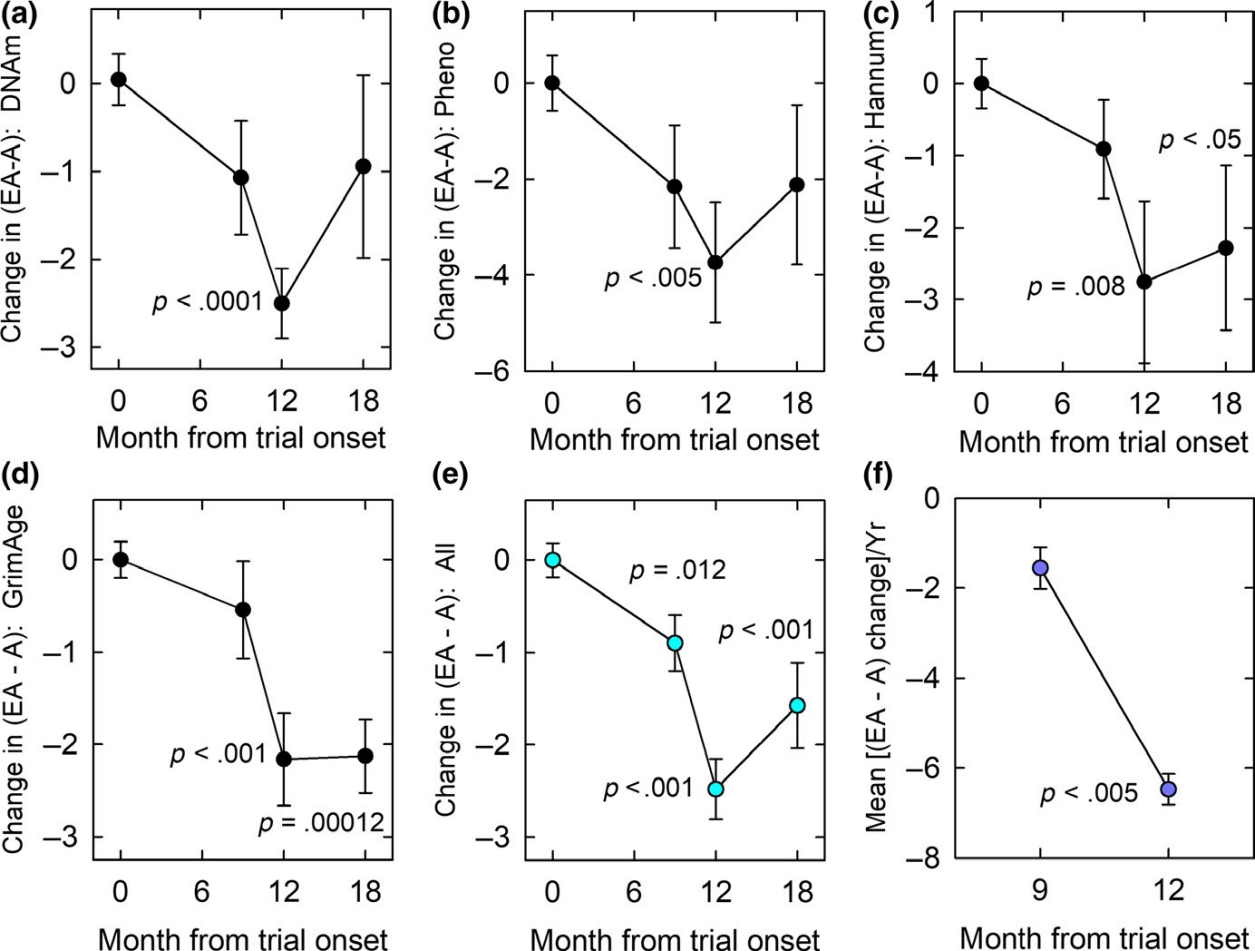
Treatment‐induced changes in epigenetic age. EA, epigenetic age; A, chronological age; changes depicted relative to EA‐A before treatment [(EA‐A)_0_]. (a) Decline in Horvath epigenetic age by 12 months of treatment. Overall, linear mixed‐model analyses (LMMAs) indicated a *P* value of .0009 over months 0–12 and .018 over months 0–18. (b) Decline in PhenoAge by 12 months of treatment. LMMA overall *P* values are .0064 for months 0–12 and .028 for months 0–18. (c) Decline in Hannum epigenetic age by 12 months, and continuing epigenetic age regression to 18 months. LMMA *p* = .012 for months 0–12 and *p* = .0092 for months 0–18. (d) Persistent decline in GrimAge age at 12–18 months. LMMA showed *p* = .0049 for months 0–12 and *p* = .0016 for months 0–18. (e) Mean of all four epigenetic aging clock results, indicating significant overall epigenetic aging regression at trial months 9–18. By LMMA, *p* = .0003 for 0–12 mo and .0016 for 0–18 mo. (f) Significant (*p* < .005) change in the rate of change of [(EA‐A)–(EA‐A)_0_] between 0–9 months of treatment (plotted at 9 months) and 9–12 months of treatment (plotted at 12 months)

Although, on average, trial volunteer epigenetic ages (EAs) were lower than their chronological ages (As) at baseline [(EA‐A)_0_ < 0, Table 1], epigenetic age was nevertheless significantly decreased by treatment based on the results of all four epigenetic clocks (Figure 5a–d), with a mean change in EA‐A after 12 months of about 2.5 years (Figure 5e). The effect of treatment on epigenetic age regression at 12 months was not dependent on age at onset of treatment or on (EA‐A)_0_ (Table 1). Linear mixed‐model analysis (LMMA) showed significance levels for individual clocks ranging from *p* = .0009–.012 over months 0–12 to *p* = .0016–.028 for months 0–18 (Figure 5 legend). Horvath clock LMMA results were unchanged (*p* = .018) after adjusting for changes in blood cell composition (lymphocyte count, percent of senescent CD8 T cells among CD8 T cells, and the LMR). Although there was a general tendency for EA‐A to trend back toward (EA‐A)_0_ 6 months after discontinuation of treatment, this trend was incomplete, leaving on average an improvement of more than 1.5 years at trial month 18 (Figure 5e, *p* < .001). In addition, the GrimAge clock, which is specifically able to predict human life expectancy (Lu et al., 2019), showed no regression of (EA‐A)‐(EA‐A)_0_ after treatment, with a gain of approximately 2.1 years at 12 months still remaining at trial month 18 (Figure 5d; Table 1). Furthermore, comparing the rates of aging regression between 0–9 and 9–12 months showed that, for every age estimator, the rate of aging regression appeared to accelerate substantially with increasing treatment time (Figure 5a–d and Table 1), with a mean slope over all four clocks of −1.56 ± 0.46 years/year in the first 9 months to −6.48 ± 0.34 years/year in the last 3 months of treatment (*p* < .005, Figure 5f).

**Table 1:** Epigenetic aging characteristics of the study population

Epigenetic clock	DNAm (Horvath)	DNAm P (Levine)	DNAm H (Hannum)	DNAm G (Lu)
(EA‐A)_0_	−3.95 ± 1.19	−17.5 ± 0.98	−12.6 ± 0.91	−2.80 ± 1.45
(EA‐A)_9_–(EA‐A)_0_	−1.071 ± 0.65	−2.16 ± 1.28	−0.91 ± 0.69	−0.54 ± 0.52
(EA‐A)_12_–(EA‐A)_0_	−2.50 ± 0.40	−3.73 ± 1.26	−2.76 ± 1.13	−2.16 ± 0.50
(EA‐A)_18_–(EA‐A)_0_	−0.94 ± 1.04	−2.12 ± 1.66	−2.28 ± 1.14	−2.12 ± 0.40
[(EA‐A)_9_–(EA‐A)_0_] per year	−1.43 ± 0.86	−2.87 ± 1.71	−1.21 ± 0.92	−0.72 ± 0.70
[(EA‐A)_12_–(EA‐A)_9_] per year	−5.74 ± 2.53	−6.31 ± 4.59	−7.38 ± 4.66	−6.48 ± 1.31
Correlation between [(EA‐A)_12_–(EA‐A)_0_] and A_0_	*r* ^2^ = .02	*r* ^2^ = .03	*r* ^2^ = .153	*r* ^2^ = .04
	*p* = .71	*p* = .68	*p* = .298	*p* = .61
Correlation between [(EA‐A)_12_–(EA‐A)_0_] and (EA‐A)_0_	*r* ^2^ = .02	*r* ^2^ = .23	*r* ^2^ = .35	*r* ^2^ = .09
	*p* = .72	*p* = .196	*p* = .096	*p* = .43

Note

EA = epigenetic age; A = chronological age; 0 = at zero months (trial onset); 9, 12, 18 = at 9, 12, and 18 months after the trial onset; A_0_ = age at trial onset; all results given in years or in years per year.

## 3 DISCUSSION

The TRIIM trial was designed to investigate the possibility of thymus regeneration and reversion of immunosenescent trends in healthy aging men while minimizing side effects and any possible risks. Our results support the feasibility of this goal but unexpectedly also bring to light robust evidence that regression of multiple aspects and biomarkers of aging is possible in man. These two observations may be related.

Thymus regeneration and reactivation by growth hormone administration have been established in aging rats and dogs by restoration of youthful thymic histology (Goff, Roth, Arp, & al., e., 1987; Kelley et al., 1986) and by reversal of age‐related immune deficits (Kelley et al., 1986). In humans, the existence of surviving thymic tissue after the age of about 54, which is required for successful thymus regeneration in older individuals, has been questioned (Simanovsky, Hiller, Loubashevsky, & Rozovsky, 2012). Available reports indicating increased thymic CT density and immunological improvements induced by rhGH in HIV patients (Napolitano et al., 2008; Plana et al., 2011), whose thymi are physiologically unusual (McCune et al., 1998), are silent on whether regeneration was observed in individuals over the age of 50. The present study now establishes highly significant evidence of thymic regeneration in normal aging men accompanied by improvements in a variety of disease risk factors and age‐related immunological parameters as well as significant correlations between TFFF and favorable changes in monocyte percentages and the LMR, independent of age up to the age of 65 at the onset of treatment. These observations are consistent with the known ability of growth hormone to stimulate hematopoiesis and thymic epithelial cell proliferation (Savino, 2007). Our finding of an increase in FGF‐21 levels after 12 months of treatment suggests that thymic regeneration by the present treatment may be mediated in part by this cytokine (Youm, Horvath, Mangelsdorf, Kliewer, & Dixit, 2016), which we believe is a novel finding.

We have not found previous studies associating rhGH administration with an increased LMR or reduced monocyte levels, whereas DHEA administration may actually increase monocyte levels (Khorram, Vu, & Yen, 1997). The mechanisms involved are not clear, but the unexpected effect of our treatment on the LMR may be of considerable significance, for two reasons.

First, higher LMRs are associated with better prognoses for a variety of leading sources of human mortality, including at least 8 types of cancer (e.g., prostate cancer; Caglayan et al., 2019), atherosclerosis (Gong et al., 2018), cardiovascular disease (Ji et al., 2017), and stroke (Ren, Liu, Wang, & Gao, 2017)], and are also associated with less generalized inflammation (Gong et al., 2018). Protection from cardiovascular disease has been associated with an LMR above 5 (Gong et al., 2018). In our study, mean volunteer LMRs were below 5 at baseline but were well above 5 at the end of treatment and 6 months after the end of treatment.

Second, the great majority of monocytes are CD38‐positive. CD38 is an NADase ectoenzyme and a degrader of the NAD^+^ precursor, nicotinamide mononucleotide, and increased CD38 expression with age appears to be the primary cause of age‐related tissue NAD^+^ depletion in mice and most likely in man as well (Camacho‐Pereira et al., 2016). Induction of CD38 with aging has been proposed to be driven by age‐related inflammation, and to originate in inflammatory cells residing in tissues (Camacho‐Pereira et al., 2016), which in principle might include monocytes. Although many other immune cells express CD38, we did not detect any other CD38^+^ immune cell population that declined in response to thymus regeneration treatment, suggesting that monocytes may be of particular significance. Our observation of a decline in CRP in combination with reduced monocyte levels therefore suggests the possibility of an increase in tissue NAD^+^ levels. The mechanism by which CRP was reduced is not clear, but reactivation of negative selection in the thymus could in theory reduce autoimmune‐related inflammation; clearance of pro‐inflammatory viruses or senescent somatic cells could also be involved. Given the relationship between age‐related tissue NAD^+^ depletion and the onset of aging phenotypes (Das et al., 2018; Poljsak & Milisav, 2016), an increased tissue NAD^+^ content could be connected to our observations of reversed epigenetic aging, to the general association of higher LMRs with better health, and to previous observations linking thymus transplantation to regression of various nonimmunological aging processes (Fabris, Mocchegiani, Muzzioli, & Provinciali, 1988). Consistent with this possibility, we observed that strong declines in total and CD38^+^ monocytes, increases in LMR, and reduced GrimAge all persisted for 6 months following discontinuation of treatment.

Treatment‐induced declines in PD‐1^+^ CD8 T cells are consistent with the possibility that thymus regeneration treatment epigenetically reprograms “exhausted” CD8 T cells (*T*_EX_), a recently characterized distinct PD‐1^+^ CD8 population (Khan et al., 2019). These cells are of great interest in part because PD‐1 is an immune checkpoint molecule that inhibits T‐cell proliferative responses (Henson, Macaulay, Franzese, & Akbar, 2012) and is involved in cancer evasion of immune control, leading to intensive efforts to develop pharmacological immune checkpoint inhibitors that target PD‐1 (Khan et al., 2019). Blocking PD‐1 signaling improves proliferation of “senescent” human CD‐8 cells (Henson, Macaulay, Riddell, Nunn, & Akbar, 2015). Mice, too, develop a subpopulation of exhausted CD8 cells with aging that are PD‐1^+^ and Tim‐3^+^ (Lee et al., 2016). Therefore, reduction of PD‐1^+^ CD8 T cells with thymus regeneration treatment likely represents a significant improvement in immune status.

Treatment‐induced increases in naïve CD4 and naïve CD8 T cells were relatively small compared to changes reported in rhGH‐treated HIV patients, but our volunteer population was pre‐immunosenescent and not depleted of naïve CD4 and naïve CD8 T cells at baseline. Positive responses also occurred despite potential complications caused by lymph node aging (Thompson et al., 2019). Therefore, the small increases observed in these cells and in CD4 T‐cell RTEs are consistent with the ultimate goal of preventing or reversing the normal age‐related collapse of the TCR repertoire at ages just above those of our study population (Naylor et al., 2005).

There may be both immunological and non‐immunological mechanisms of epigenetic aging reversal. GH, DHEA, and metformin have unique effects that are in opposition to aging, and it is possible that the specific combination of these agents activates a broad enough range of therapeutic pathways to account for the previously unpredictable reversal of epigenetic aging, even independently of the immunological markers we have measured.

In this regard, it must be pointed out that GH and IGF‐1 can also have pro‐aging effects and that most gerontologists therefore favor reducing rather than increasing the levels of these factors (Longo et al., 2015). However, most past studies of aging and GH/IGF‐1 are confounded by the use of mutations that affect the developmental programming of aging, which is not necessarily relevant to nonmutant adults. For example, such mutations in mice alter the normal innervation of the hypothalamus during brain development and prevent the hypothalamic inflammation in the adult (Sadagurski et al., 2015). Hypothalamic inflammation may program adult body‐wide aging in nonmutants (Zhang et al., 2017), but it seems unlikely that lowering IGF‐1 in normal non‐mutant adults can provide the same protection. A second problem with past studies is a general failure to uncouple GH/IGF‐1 signaling from lifelong changes in insulin signaling. Human longevity seems more consistently linked to insulin sensitivity than to IGF‐1 levels, and the effects of IGF‐1 on human longevity are confounded by its inverse proportionality to insulin sensitivity (Vitale, Pellegrino, Vollery, & Hofland, 2019). We therefore believe our approach of increasing GH/IGF‐1 for a limited time in the more natural context of elevated DHEA while maximizing insulin sensitivity is justified, particularly in view of the positive role of GH and IGF‐1 in immune maintenance, the role of immune maintenance in the retardation of aging (Fabris et al., 1988), and our present results.

Whatever the mechanism of epigenetic age reversal may be, the four selected epigenetic clocks, despite measuring somewhat different features of aging and correlating differently with blood composition and leukocyte telomere length, all showed significant regression of epigenetic age. There was also a marked acceleration of epigenetic aging reversal after 9 months of treatment. The implications of the latter observation remain to be explored. Further, although epigenetic aging reversal appeared to partially regress following discontinuation of treatment according to some epigenetic clocks, this was not true for the GrimAge clock, which best predicts human life expectancy and health span (Lu et al., 2019). It remains to be seen whether follow‐up measurements of epigenetic aging using the GrimAge clock will show a persistent 2‐year gain in predicted life expectancy or a gradual loss of increased life expectancy compared to baseline. In the latter event, it will be interesting to determine whether repetition or prolongation of the trial treatment might restore or further augment the predicted lifespan gain.

Although epigenetic age does not measure all features of aging and is not synonymous with aging itself, it is the most accurate measure of biological age and age‐related disease risk available today. This justifies the use of epigenetic clocks to estimate the effectiveness of putative aging interventions on a practical timescale. The present study strongly supports this approach, having demonstrated regression of epigenetic age with high statistical significance even in a one‐year pilot trial involving only 9 volunteers. However, it will be necessary to verify the present results by replicating them in an appropriately powered follow‐up study. Although much more remains to be done, the general prospects for meaningful amelioration of human aging appear to be remarkably promising.

## 4 EXPERIMENTAL PROCEDURES

### 4.1 Volunteer recruitment and screening

Ten nominally healthy adult men from 51–65 years of age were recruited for the study by word of mouth following public announcements of the objectives of the trial. There were two cohorts, the first consisting of seven men and the second comprising three men. Cohort 1 was treated from October of 2015 to October of 2016, and cohort 2 was treated from April 2016 to April 2017. Volunteers first completed an online screening questionnaire, and qualifying candidates then provided a blood sample for objective confirmation of health eligibility. Candidates passing the second screen attended a meeting to enable physical evaluation, verification of ability to self‐inject rhGH, baseline magnetic resonance (MR) imaging of the thymus, two additional blood collections (see Appendix S2), informed consent, and instruction on how to fill out provided medication diaries intended to remind volunteers of dosing schedules and to report any adverse effects or any irregularities in dosing.

### 4.2 Trial conduct

The TRIIM trial was carried out after approval of the study protocol by the Aspire Institutional Review Board (Santee, California) and under the auspices of IND 125,851 from the Food and Drug Administration. Volunteers received MRI examinations at the Lucas Center for Imaging, blood collections at the Stanford Blood Center, and CyTOF analysis by the Human Immune Monitoring Center (HIMC) under the approval of the Stanford University Research Compliance Office. The trial was conducted consistently with the Declaration of Helsinki, Protection of Human Volunteers (21 CFR 50), Institutional Review Boards (21 CFR 56), and Obligations of Clinical Investigators (21 CFR 312). It was, in accordance with FDA guidelines for pre‐Phase I exploratory studies (https://prsinfo.clinicaltrials.gov/ACT_Checklist.pdf), not preregistered on clinicaltrials.gov.

During the first week of the trial, rhGH alone (0.015 mg/kg) was administered to obtain an initial insulin response, and during the second week, rhGH was combined with 50 mg DHEA to evaluate insulin suppression by DHEA alone. During the third week, the same doses of rhGH and DHEA were combined with 500 mg metformin. Beginning at the fourth week, all doses were individualized based on each volunteer's particular responses. Thereafter, blood was collected one week prior to trial months 2, 3, 4, 6, and 9 to enable further dose adjustments at those time points (to maximize IGF‐1 and minimize insulin), and additional blood samples were obtained at 12 months to conclude the treatment monitoring period. Dosing compliance was verified by the response of IGF‐1, DHEAS, and insulin to administration of rhGH, DHEA, and metformin, respectively; by frequent communication with trial volunteers; and by retrospective review of returned medication diaries. Additional follow‐up blood testing was done at 18 months for cohort 1; cohort 2 was not available. In selected cases, as deemed useful, supplemental blood sampling was carried out at other times.

Serum and peripheral blood mononuclear cells (PBMCs) were cryopreserved at the SBC, in the latter case using 10% dimethyl sulfoxide and fetal bovine serum (FBS), and stored there and then at the HIMC until the end of the trial to enable simultaneous CyTOF analysis and further distribution of some samples to other centers. Complete blood counts were performed by Quest Diagnostics to enable correction for cell losses caused by freezing and thawing or other factors, but in general, the need for correction was avoided by reporting cell subsets as percentages of their reference populations (e.g., naïve CD4 cells as a percent of total CD4 cells) and by gating to intact viable singlets. Other blood assays are described in Appendix S2.

rhGH (Omnitrope, Sandoz) was provided to trial volunteers and was self‐administered 3–4 times per week, depending on side effects, at bedtime along with other study medications. All volunteers were also provided with and asked to take supplements of 3,000 IU vitamin D3 and 50 mg of elemental zinc daily.

### 4.3 MR imaging and analysis

All imaging scans were performed on the same 3T GE Premier MRI scanner at the Lucas Center for Imaging at Stanford University. Standardized methods for quantitatively determining thymic fat content have not been previously described, so we employed a computational methodology that has previously been applied to the analysis of bone marrow fat content (Hu, Nayak, & Goran, 2011). These methods for quantifying fat have been shown to be more accurate than standard histopathological assessment by biopsy (Fischer et al., 2012) and provide the thymic fat fraction (TFF) as a number from 0% to 100%. TFF was determined in three replicate central thymic regions at each tested time point and was used to compute the thymic fat‐free fraction (TFFF) as 100% ‐ TFF. The same methods were also applied to determine the sternal bone marrow fat‐free fraction (BMFFF). For more details, see Appendix S2.

### 4.4 Immunophenotyping

Cryopreserved PBMCs were thawed in warm media, washed twice, and resuspended in CyFACS buffer (PBS supplemented with 2% BSA, 2 mM EDTA, and 0.1% sodium azide). Viability was determined by trypan blue dye exclusion (Vi‐CELL XR assay, Beckman Coulter Life Sciences). Cells were added to a V‐bottom microtiter plate (1.5 × 10^6^ viable cells/well) and washed once by pelleting and resuspension in fresh CyFACS buffer. The cells were stained for 60 min on ice with 50 μl of a cocktail of heavy metal isotope‐labeled antibodies directed against 35 cell surface markers (for details, and for details on cell washing, staining, permeabilization, and gating, see Appendix S2).

### 4.5 Determination of epigenetic age

The state of genomic DNA methylation was determined in previously cryopreserved PBMCs at the Centre for Molecular Medicine and Therapeutics at the University of British Columbia. Genomic DNA was extracted with the DNeasy Blood & Tissue Kit (Qiagen, Hilden, Germany), and bisulfite conversion of extracted DNA was performed using a Zymo EZ DNA Methylation Kit (Zymo Research, Irvine, CA). DNA methylation (DNAm) analysis was performed using the Illumina Infinium MethylationEPIC BeadChip (Illumina, San Diego, CA), which measures single‐CpG resolution DNAm levels at 866,836 CpG sites in the human genome. Methylation at specific sites was calculated as β = Max(M,0)/[Max(M,0) + Max(U,0)], where Max(M,0) is the fluorescence intensity of methylated (M) alleles (signal A) and Max(U,0) is the fluorescence of un‐methylated (U) alleles (signal B). Thus, β values range from 0 (completely un‐methylated) to 1 (completely methylated) (Dunning, Barbosa‐Morais, Lynch, Tavare, & Ritchie, 2008).

The specific epigenetic “clocks” chosen for use in this study were those derived by Horvath (2013; DNAm age), Hannum et al. (2013; DNAm age H), Levine et al. (2018; DNAm PhenoAge), and Lu et al. (2019; DNAm age G or “GrimAge”). The specific rationales for these choices are based on Horvath and Levine (2015), Marioni et al. (2015), and Triche, Weisenberger, van den Berg, Laird, and Siegmund, (2013) and are described in Appendix S2.

We calculated the effects of intervention on epigenetic age by first fitting all time point data to a linear mixed‐effects model for longitudinal data (multiple blood draws from the same person, adjusted for the initial DNAm age) to follow global epigenetic aging trends. Second, to isolate more detailed individual‐ and time‐specific changes, we calculated the change in epigenetic versus chronological age for each volunteer as (EA‐A) – (EA‐A)_0_ as described in the text, where EA is the epigenetic age at chronological age A and (EA‐A)_0_ is the baseline difference between EA and A.

### 4.6 Statistical analysis

We used linear mixed‐effects regression to properly account for the longitudinal nature of the information (multiple draws from the same person). When correction for time was not necessary, we used ordinary linear or nonlinear regressions. For comparisons between pretreatment and specific post‐treatment endpoints, we employed the *t* test, the *t* test for paired comparisons, or alternatives selected by SigmaPlot when the assumptions underlying *t* tests were not justified. To enable normalized comparisons to month zero baselines, replicate results for each volunteer at time zero were averaged and each replicate result was divided by the mean to produce a population of departures from 100% that could then be used for comparison against later time points. Reported *P* values do not correct for multiple comparisons because independent hypothesis testing was not carried out in most cases, and in the case of both multiple tests for epigenetic age and significant differences in CyTOF results, all significant test results were highly correlated, making Bonferroni correction overly conservative.

## CONFLICT OF INTEREST

GMF, RTB, JPW, and SH are shareholders in or have options to purchase shares in Intervene Immune, Inc., GMF and RTB are officers of Intervene Immune and are named in a related Intervene Immune patent application. All other authors declare no competing interests.

## AUTHOR CONTRIBUTIONS

GMF designed and directed the study, compiled the data for all graphics, prepared the graphics, and wrote the manuscript. RTB managed the study; contributed to its design; obtained digital data for all CyTOF, TFFF, and BMFFF analyses; and contributed to the manuscript. JPW served as the study physician, providing medical oversight, support, advice, and volunteer evaluation, and assisted with trial events. ZG designed and arranged for the antibody panel for CyTOF analysis, directed the CyTOF analyses, and assisted with trial events. SSV designed the TFFF and BMFFF measurement techniques. HM and ML provided guidance concerning correct processing and interpretation of CyTOF data and reviewed and approved our CyTOF methodology. DTSL and MSK performed all epigenetic measurements and provided helpful insights for the manuscript. SH carried out all epigenetic clock calculations, performed all linear mixed‐model correlations, and contributed to the manuscript. All authors approved the manuscript.
