# Supplementary material for: Reversal of epigenetic aging and immunosenescent trends in humans
Source: Aging Cell. 2019 Sep 8;18(6):e13028. doi: 10.1111/acel.13028 (PMC6826138; doi:10.1111/acel.13028)

Supplemental Table

| Table S1: Persistence of Monocyte Declines after the End of Treatment | |
| --- | --- |
| Normalized Monocytes at time zero | Normalized Monocytes at 18 months |
| 1.345 | 0.693 |
| 0.655 | 1.071 |
| 1.011 | 0.414 |
| 0.989 | 0.971 |
| 1.026 | 0.353 |
| 0.974 | 0.470 |
| 1.365 |  |
| 0.635 |  |
| 0.997 |  |
| 1.003 |  |
| 1.286 |  |
| 0.714 |  |
|  |  |
| Mean: 1.00 | Mean: 0.662 |
| Std Dev: 0.247 | Std Dev: 0.303 |
| SEM: 0.071 | SEM: 0.124 |
|  | t=2.546 |
|  | p= 0.0216 |

Legend to Supplemental Figure S1

Fig. S1. General trend for CD4 T cell recent thymic emigrant percentages (CD4 RTEs) to increase by a median of about 20% (and by a maximum of ~2-fold) by month 12 of treatment in comparison to percentages at baseline (CD4 RTEs)_0_. Green points: means and SEMs, displaced by 0.5 months for greater visibility.

Supplemental Figure 1


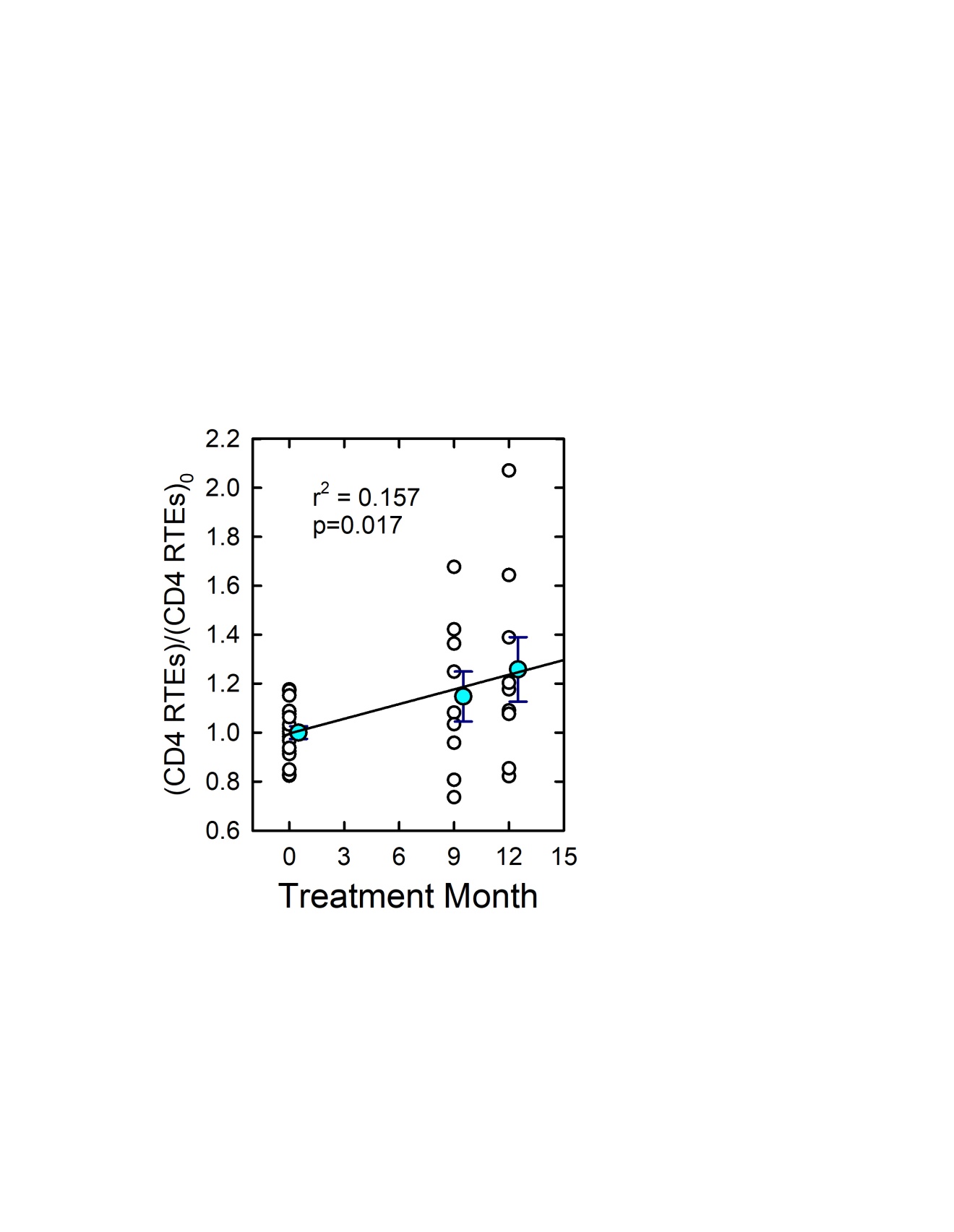


Legend to Supplemental Figure S2

Fig. S2. Relative increase in serum FGF-21 induced by 12 months of thymus regeneration treatment.

Supplemental Figure S2


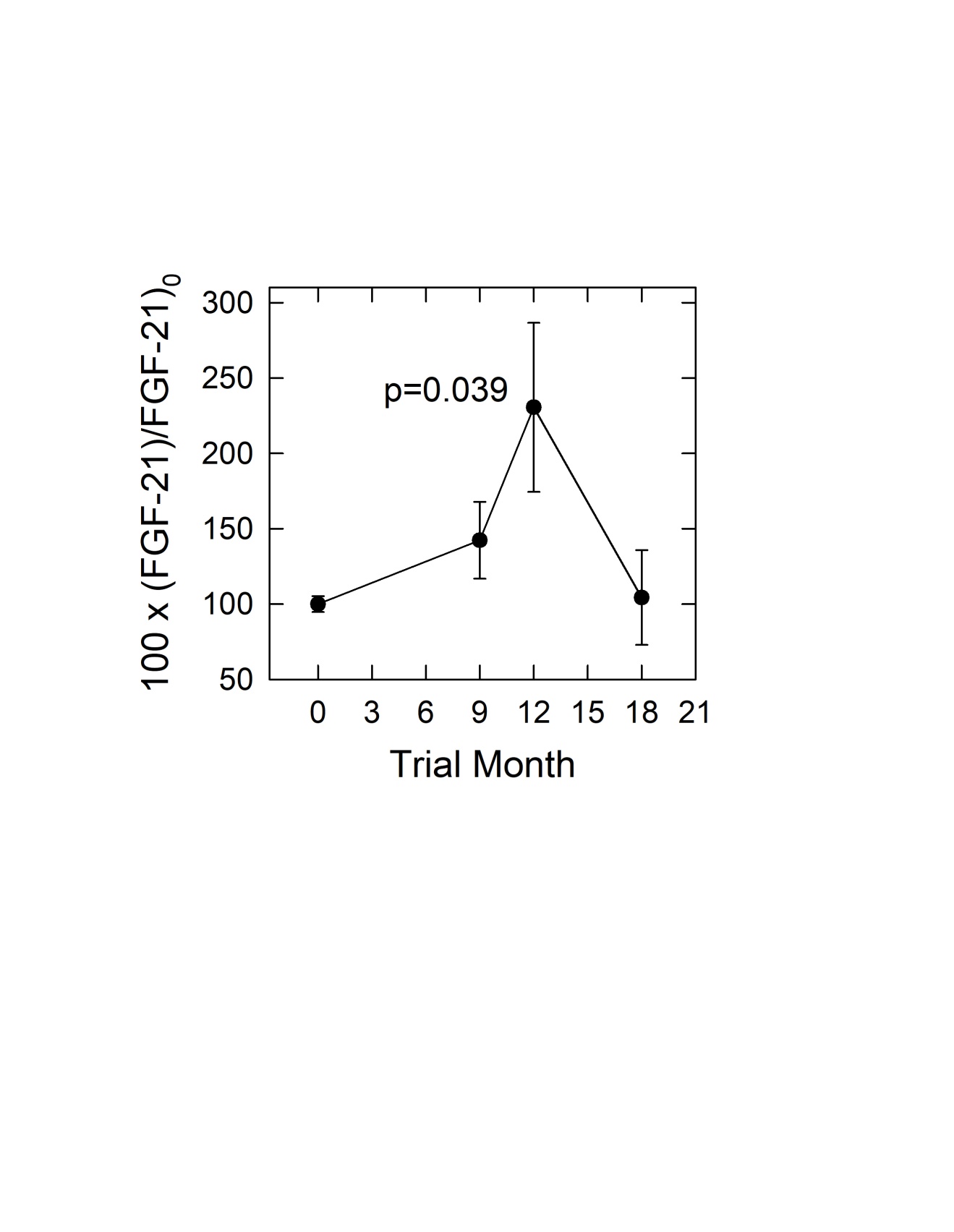

Supplement: Supplementary file 1 [file ACEL-18-e13028-s001.docx]
