# Supplementary material for: Reversal of epigenetic aging and immunosenescent trends in humans
Source: Aging Cell. 2019 Sep 8;18(6):e13028. doi: 10.1111/acel.13028 (PMC6826138; doi:10.1111/acel.13028)
Supplement: Supplementary file 2 [file ACEL-18-e13028-s002.docx]

**Appendix S2**

Other blood tests and biomarkers and blood testing mechanics

Routine blood tests were performed through Quest Diagnostics to monitor protocol endpoints and safety, including tests for insulin, glucose, IGF-1, IGF1BP3, IL-6, C-reactive protein (CRP), DHEA, DHEAS, testosterone, serum creatinine, blood lipids, comprehensive metabolic panel metabolites and electrolytes, creatine kinase, HIV, CMV, and EBV anti-viral antibodies, and other analytes. Estimated glomerular filtration rate (eGFR) was calculated as 186 x (Cr/88.4)^‑1.154^ x Age^‑0.203^, where Cr is the serum creatinine concentration in μmoles/liter and Age is the subject’s age in years. CRP (high-sensitivity test, hsCRP) and IL-6 levels were interpreted as biomarkers of systemic inflammation, and the Quest Diagnostics lymphocyte subset panel 4 was used to follow CD4 and CD8 immune cell subset changes during the course of the study. Complete blood counts were obtained periodically from Quest and the SBC. ELISA assays for FGF-21 (from R&D Systems) were carried out by Gene and Cell Technologies, Inc. Other tests that did not result in significant differences are not reported here.

Baseline blood samples were collected approximately 28 hours apart (the first a morning fasting collection and the second an early afternoon postprandial collection) for intentionally redundant determination of baseline T cell populations by CyTOF analysis (see below). These blood collections and blood collections at 9 and 12 months, which were also intended for CyTOF evaluation, were done at the Stanford Blood Center (SBC). For blood tests at other time points, blood was collected at Quest Diagnostics laboratories located near each volunteer. All enrolled candidates were negative for CMV, EBV, HSV, and HIV.

Trial exclusion criteria

Exclusion criteria included having diabetes, a body mass index of over 30, impaired renal or hepatic function, elevated general cancer risk (as evidenced by a prior history of cancer or a familial history of cancer), PSA above the normal range, unresponsive benign prostatic hypertrophy, elevated hsCRP or IL-6 levels, prior use of GH or evidence of GH resistance, HIV infection, or other unstable medical conditions. Alcoholism, drug addition, and an inability to provide informed consent and to follow trial instructions were also grounds for exclusion.

TFFF determination

LAVA Flex algorithms provide a 3D FSPGR T1-weighted technique that generates four image sets in a rapid acquisition time of 15-25 seconds. We used a fast two-point Dixon method to generate fat-only and water-only images (fat-suppressed images) in addition to T1-weighted in-phase and out-of-phase images. This enables co-registered images to be obtained within the same breath hold that enable the identification of fatty thymic tissue as well as dense functional thymic tissue. To compute treatment-induced changes in TFFF, T1-weighted in-phase and out-of-phase images were first used to identify regions of thymic tissue, and comparable regions were then analyzed in images obtained before, during, and after treatment. Due to the complexity of thymic morphology and uncertainties in identifying the thymic boundary in some areas, we limited our analyses to the determination of thymic fat content only within well-defined central thymic regions and did not attempt to monitor changes in total thymic volume. The regions of interest were defined on sequential axial sections located between the sternal notch and the base of the heart (Napolitano et al. 2008).

Immunophenotyping

The specific assignment of metal isotopes to antibodies directed against different cell surface markers was as follows: ^150^Nd-CD3, ^143^Nd-CD4, ^144^Nd-CD8, ^142^Nd-CD11a, ^153^Eu-CD11b, ^148^Nd-CD11c, ^154^Sm-CD14, ^164^Dy-CD20, ^168^Er-CD24, ^176^Yb-CD25, ^152^Sm-CD27, ^167^Er-CD28, ^145^Nd-CD31, ^166^Er-CD33, ^151^Eu-CD38, ^162^Dy-CD45RA, ^149^Sm-CD45RO, ^141^Pr-CD49d, ^174^Yb-CD56, ^113^In-CD57, ^147^Sm-CD85j, ^157^Gd-CD86, ^156^Gd-CD94, ^170^Er-CD122, ^173^Yb-CD123, ^165^Ho-CD127, ^172^Yb-PD-1, ^175^Lu-HLA-DR, ^171^Yb-TCRgd, ^159^Tb-CXCR3, ^158^Gd-CXCR5, ^155^Gd-CCR6, ^160^Gd-CCR7, ^161^Dy-PTK7, ^146^Nd-IgD, and ^169^Tm-ICOS. All antibodies were from purified unconjugated, carrier-protein-free stocks (BD Biosciences, BioLegend, or R&D Systems). The polymer and metal isotopes were from DVS Sciences. Antibody-labeled cells were washed twice by pelleting and resuspension with 250 μL FACS buffer and then further marked for viability by resuspension in 100 μL PBS buffer containing 2 μg/mL of Live-Dead (maleimido-monoamide-DOTA containing natural-abundance indium (^115^I), from Macrocyclics, Inc.; assay B-272) according to manufacturer instructions. After indium labeling, the cells were washed twice by pelleting and resuspension with 250 µL PBS, and then resuspended in 100 µL 2% PFA in PBS and placed at 4°C overnight. The next day, the cells were pelleted, resuspended in fresh PBS, pelleted again, resuspended in 100 µL of eBiosciences permeabilization buffer (1x in PBS), and placed on ice for 45 min before washing twice with 250 µL PBS. Intact cells were labeled by transferring them into 100 µL of ^191^Ir-containing DNA intercalator (1:2000 dilution in PBS; DVS Sciences) and incubation at room temperature for 20 min. After washing twice in 250 µL Milli-Q water, the cells were diluted in a total volume of 700 µL of Milli-Q water before injection into the CyTOF apparatus (DVS Sciences).

Data analysis was performed using Cytobank by gating first on intact cells based on the iridium isotope from the intercalator, second on singlets by iridium signal vs cell length, and third on viable cells as judged by indium exclusion based on the Indium-LiveDead assay, followed by cell subset-specific gating using FlowJo (FlowJo, LLC, Ashland, OR). The CITRUS software package (Cytobank, Inc., Santa Clara, CA) was used to provide initial indications of CyTOF-identifiable changes in immune cell clusters (Spitzer et al. 2015), but all final statistical tests were carried out on FlowJo raw data for specific markers and marker combinations.

Choice of epigenetic aging clocks and related methods

As our primary epigenetic biomarker of aging, we used the pan tissue DNAm age estimator of Horvath (Horvath 2013) based on 353 CpGs for three reasons. First, the resulting age estimate (DNAm age), correlates strongly with chronological age in multiple cell types (CD4 T cells, monocytes, B cells, glial cells, and neurons) as well as in tissues and organs, including: whole blood, brain, breast, kidney, liver, lung, and saliva (Horvath 2013). Second, the pan tissue estimator is less confounded by changes in blood cell composition than other published estimators (Horvath and Levine 2015; Marioni et al. 2015). Third, DNAm age has been shown to be more accurate than chronological age for determining the biological age of individuals (Horvath 2013). To confirm and test the robustness of our Horvath age estimator results, we compared them to epigenetic age estimates obtained using the following alternative DNAm based biomarkers: the epigenetic clock for blood samples by Hannum (Hannum et al. 2013) (DNAm age H), the phenotypic age estimator (DNAm PhenoAge) by Levine (Levine et al. 2018), and the recently described “GrimAge” clock that predicts the age of death of tested individuals (DNAm age G; (Lu et al. 2019)). Unlike the pan tissue clock by Horvath, the Hannum, PhenoAge, and GrimAge clocks exhibit moderately high correlations with blood cell counts (e.g., they correlate negatively with naïve CD8 T cells) as reviewed elsewhere (Horvath and Raj 2018).

We used the Noob normalization method (Triche et al. 2013) implemented in the "Minfi" R package. Epigenetic age was calculated from the Illumina Infinium EPIC array methylation state profiles.
